# Avoiding Parotid Gland Injury During Lateral Cheek Cogged Thread Lifting (Sihler Thread): Cadaveric Study

**DOI:** 10.1111/jocd.71009

**Published:** 2026-07-02

**Authors:** Gi‐Woong Hong, Kyu‐Ho Yi

**Affiliations:** ^1^ Samskin Plastic Surgery Clinic Seoul Korea; ^2^ You and I Clinic Seoul Korea

## 1 Introduction

Thread‐based lifting with barbed or cogged sutures was developed as a minimally invasive alternative to rhytidectomy and has evolved into multiple absorbable and nonabsorbable systems, insertion techniques, and vector designs [1, 2, 3]. The procedure is generally performed in a subcutaneous, SMAS‐adjacent, or selectively sub‐SMAS plane to reposition ptotic soft tissue along planned lifting vectors. Compared with open facial rejuvenation surgery, thread lifting offers shorter recovery and lower procedural invasiveness, but it also has a distinct complication profile [4, 5, 6, 7].

Common adverse events include swelling, bruising, dimpling, contour irregularity, thread palpability, and thread extrusion [4, 5, 6, 7]. Less frequent but clinically consequential complications include facial nerve injury and salivary system injury. Parotid gland or duct injury may present as focal swelling, sialocele, salivary fistula, meal‐related pain, or ductal obstruction, and may require aspiration, compression, botulinum toxin injection, sialendoscopic treatment, or surgical management [5, 8, 9].

The lateral cheek is an anatomically complex region for thread lifting because commonly used vectors traverse the preauricular and lateral cheek area, where the parotid gland, masseter muscle, retaining ligaments, SMAS, parotid‐masseteric fascia, and facial nerve branches are closely related. Classic and modern anatomical studies have shown that retaining ligaments and facial spaces define predictable tissue boundaries and zones of adherence [10, 11, 12, 13, 14]. These structures provide important landmarks for safe dissection in facelift surgery and may also explain why resistance is encountered during blind cannula passage in minimally invasive thread procedures.

Despite increasing recognition of thread‐lifting complications, the specific anatomical mechanism of parotid gland injury remains insufficiently characterized. Existing reports identify salivary complications as rare but clinically important events, yet few studies directly correlate lateral cheek thread trajectories with cadaveric anatomy of the parotid‐masseteric fascia, parotid capsule, and sub‐SMAS spaces. As a result, the distinction between safe superficial or SMAS‐adjacent passage and potentially hazardous deep fascial violation is not always clearly defined in the thread‐lifting literature.

This anatomical technical note synthesizes relevant lateral cheek anatomy, correlates the literature with cadaveric demonstrations, and proposes a practical mechanism by which parotid gland injury may occur during lateral cheek cogged thread lifting. In response to the need for clinically translatable guidance, the article also introduces a plane‐control framework, discusses the tradeoffs between superficial and deeper thread placement strategies, integrates ultrasound‐based safety concepts, and proposes a stepwise approach for suspected parotid violation. The aim is to provide anatomy‐based guidance for reducing salivary injury risk while preserving clinically effective lifting vectors.

## 2 Materials and Methods

This study was designed as an anatomical technical note integrating a targeted narrative literature review with illustrative cadaveric anatomical correlation. The purpose was not to perform a systematic review, estimate complication incidence, or quantify anatomical variation, but to synthesize relevant lateral cheek anatomy and illustrate a plausible mechanism of parotid gland injury during cogged thread lifting.

A targeted literature review was performed focusing on facial layers, retaining ligaments, superficial and deep facial fasciae, fat compartments, sub‐SMAS facial spaces, thread‐lifting techniques, thread‐lifting complication patterns, salivary complications after facial esthetic procedures, and ultrasound‐based assessment of facial soft tissue and salivary structures. Literature was prioritized if it addressed: (i) facial layers, retaining ligaments, fat compartments, and facial spaces relevant to the lateral cheek; (ii) thread‐lifting techniques and complication patterns, including systematic reviews and large complication series; (iii) salivary complications after facial esthetic procedures; or (iv) imaging concepts relevant to identifying the parotid gland, parotid duct, tissue thickness, fluid collection, or misplaced threads.

Cadaveric dissection photographs and schematic figures were reviewed to correlate literature‐based anatomy with the proposed mechanism of parotid injury. The cadaveric material was used illustratively to demonstrate the relationship among the SMAS, retaining ligaments, parotid‐masseteric fascia, parotid capsule, parotid gland, parotid duct, and facial nerve branches in the lateral cheek. Because the available cadaveric component was not designed as a quantitative anatomical series, the observations were interpreted as demonstrative anatomical correlation rather than as prevalence data. The figures were selected to demonstrate four anatomical concepts: first, the relationship between retaining ligaments, SMAS thickness, and expected zones of resistance; second, the protective role of the parotid‐masseteric fascia and parotid capsule; third, the distinction between superficial, SMAS‐adjacent, and hazardous deep trajectories; and fourth, the proposed mechanism by which deep cannula passage may breach these fascial boundaries and enter the parotid gland.

Observations from cadaveric demonstrations were interpreted in relation to previously published anatomical and clinical literature. Particular attention was given to whether the illustrated relationships were consistent with established descriptions of the lateral cheek SMAS, retaining ligaments, preparotid and premasseteric spaces, parotid‐masseteric fascia, and parotid capsule. Because this study was anatomical and illustrative, no statistical comparison, quantitative anatomical measurement, or clinical outcome analysis was performed. Anatomical variability in the anterior extent of the parotid gland, SMAS thickness, retaining ligament density, and soft‐tissue thickness was therefore considered in the interpretation rather than quantified.

## 3 Results: Literature‐Based Anatomy and Cadaveric Correlation

For procedural interpretation, the lateral cheek should be conceptualized as a layered region rather than as a uniform soft‐tissue plane. A superficial subcutaneous corridor lies within the fat superficial to the SMAS and generally remains distant from the parotid capsule. A SMAS‐adjacent corridor lies immediately superficial to or closely related to the SMAS and may provide stronger mechanical coupling while still respecting the deeper parotid‐masseteric fascia. By contrast, a hazardous deep trajectory is one in which the cannula or thread violates the parotid‐masseteric fascia and parotid capsule, allowing possible intraparotid passage. This framework is used below to interpret the cadaveric demonstrations and to translate the anatomical findings into procedural guidance.

The key anatomical layers and structures relevant to plane control and parotid safety are summarized in Table 1.

**TABLE 1:** Key anatomical layers and structures relevant to plane control and parotid safety during lateral cheek cogged thread lifting.

Layer/structure	Role in thread lifting	Plane‐control relevance	Why it matters for parotid safety	Practical pearl
Superficial subcutaneous fat	Common corridor for many thread designs and superficial lifting vectors	Represents the superficial subcutaneous corridor; generally remains above the SMAS and away from the parotid capsule	Too superficial may increase dimpling, visibility, palpability, or contour irregularity; however, it usually provides greater distance from the parotid gland than deeper trajectories	Maintain smooth cannula movement and avoid skin tethering; if dimpling occurs, reassess whether the pass is too superficial
SMAS of the lateral cheek	Provides a fibrous structure that may improve mechanical coupling and lifting effect	Defines the SMAS‐adjacent corridor when the thread remains immediately superficial to or closely related to the SMAS without crossing the parotid‐masseteric fascia	Perforation through the SMAS or uncontrolled deep passage may allow the cannula to enter a hazardous deeper plane	Treat increased resistance as a warning sign; withdraw and redirect rather than forcing the cannula forward
Retaining ligaments and dense septal zones	Transmit traction and define zones of adherence along lateral cheek lifting vectors	These fixed structures may create predictable resistance points and may deflect the cannula from the intended plane	Forceful advancement across ligamentous resistance may contribute to loss of plane control and deeper‐than‐intended passage	Pause, reassess, and redirect when fixed resistance is encountered
Parotid‐masseteric fascia and parotid capsule	Form the protective deep boundary superficial to the parotid gland	Crossing these structures converts a controlled superficial or SMAS‐adjacent trajectory into a hazardous deep trajectory	Breach of the fascia and capsule may permit intraparotid thread passage, with risk of swelling, sialocele, salivary fistula, ductal symptoms, or gland injury	Regard these structures as “do‐not‐cross” safety boundaries
Parotid gland, parotid duct region, and facial nerve branches	Structures at risk when the thread is placed too deeply in the lateral cheek or anterior parotid region	These structures lie deep to the intended safe corridors and become relevant when the protective fascial boundary is violated	Ductal injury may cause meal‐related swelling, obstruction, stenosis, or sialocele; nerve irritation or injury may cause weakness or sensory symptoms	Consider ultrasound assessment when postoperative swelling is focal, progressive, recurrent, or meal‐related

Abbreviation: SMAS, superficial musculoaponeurotic system.

Retaining ligaments and SMAS thickness define corridors and resistance points for thread passage.

### 3.1 Retaining Ligaments and SMAS Thickness Define Corridors for Thread Passage

Published anatomical studies show that retaining ligaments tether the SMAS and superficial fat compartments to the skeleton and deep fascia, thereby segmenting the face into functional units and facial spaces [10, 11, 14]. The zygomatic ligament complex is particularly relevant to lateral cheek lifting because it influences both tissue mobility and the transmission of traction along lifting vectors. These ligamentous structures may also correspond to areas where increased resistance is encountered during cannula passage.

**FIGURE 1:**
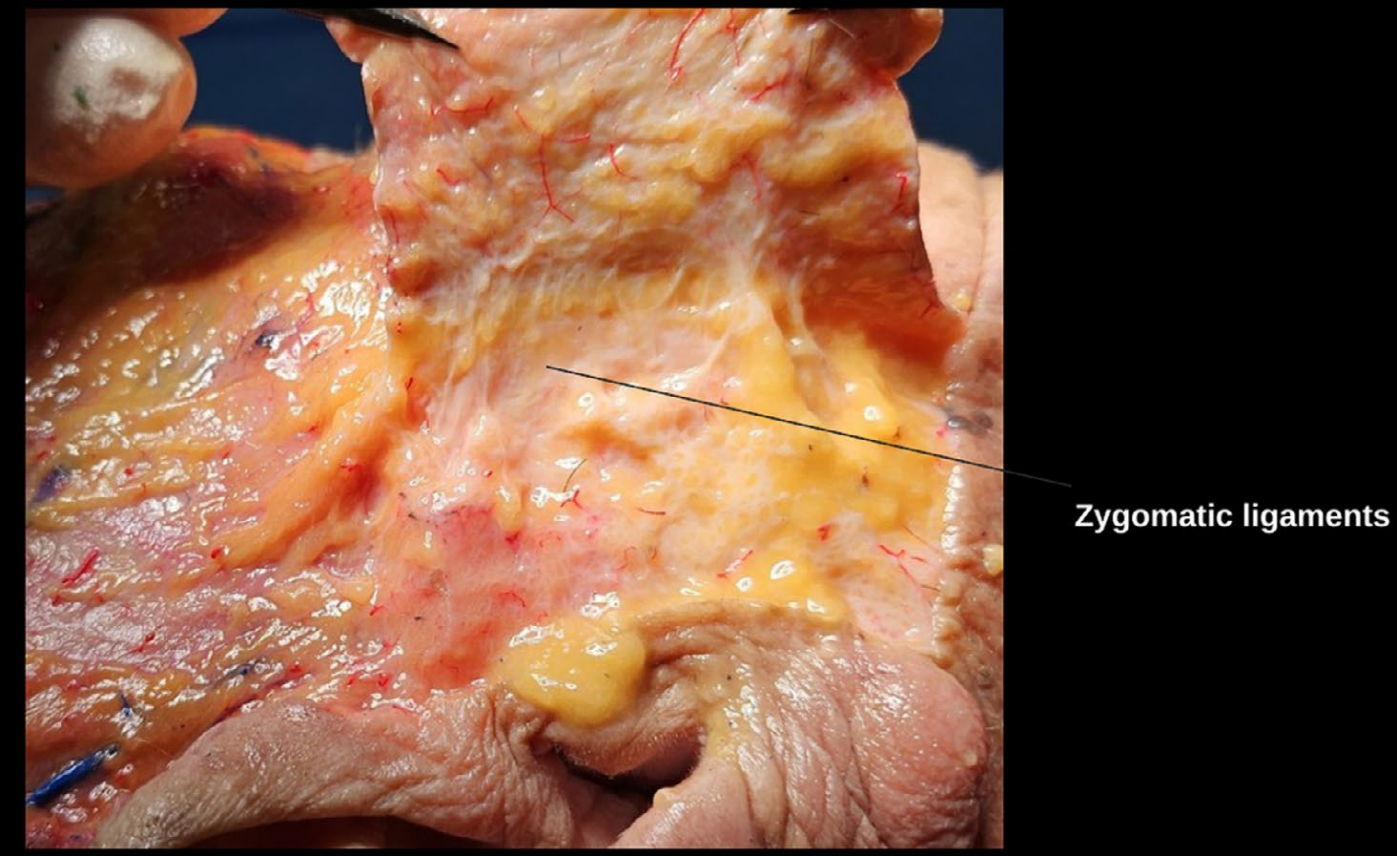
Cadaveric photograph demonstrating the zygomatic retaining ligaments over the zygomatic arch. These ligamentous structures are relevant to lateral cheek lifting vectors because they represent zones of adherence where cannula resistance may be encountered.

The lateral cheek SMAS is generally thicker and more fibrous than the medial cheek SMAS, providing a potential structure for mechanical coupling during thread lifting [15, 16]. However, this same fibrous quality may increase the risk of cannula deflection or unintended change in depth if the operator advances against resistance without reassessing the plane. In the cadaveric demonstration, the zygomatic retaining ligaments and lateral cheek SMAS illustrate why the lateral cheek should not be treated as a uniform soft‐tissue plane during thread insertion (Figure 1). Procedurally, resistance in this region should be interpreted as a cue to pause, withdraw slightly, reassess the depth, and redirect within the intended superficial or SMAS‐adjacent corridor rather than force the cannula forward.

### 3.2 The Parotid‐Masseteric Fascia and Parotid Capsule Act as Protective Deep Boundaries

The parotid gland is enclosed by a fascial capsule derived from the deep cervical fascia and is closely related anteriorly to the parotid‐masseteric fascia overlying the masseter muscle. In the lateral cheek, this fascia forms an important deep boundary for safe superficial or SMAS‐adjacent passage [12, 13, 15]. When the cannula and thread remain superficial to the parotid‐masseteric fascia or within a controlled plane superficial to the gland, the parotid parenchyma, intraparotid facial nerve branches, and parotid duct are separated from the thread trajectory by intervening fascial structures [12, 13, 17].

**FIGURE 2:**
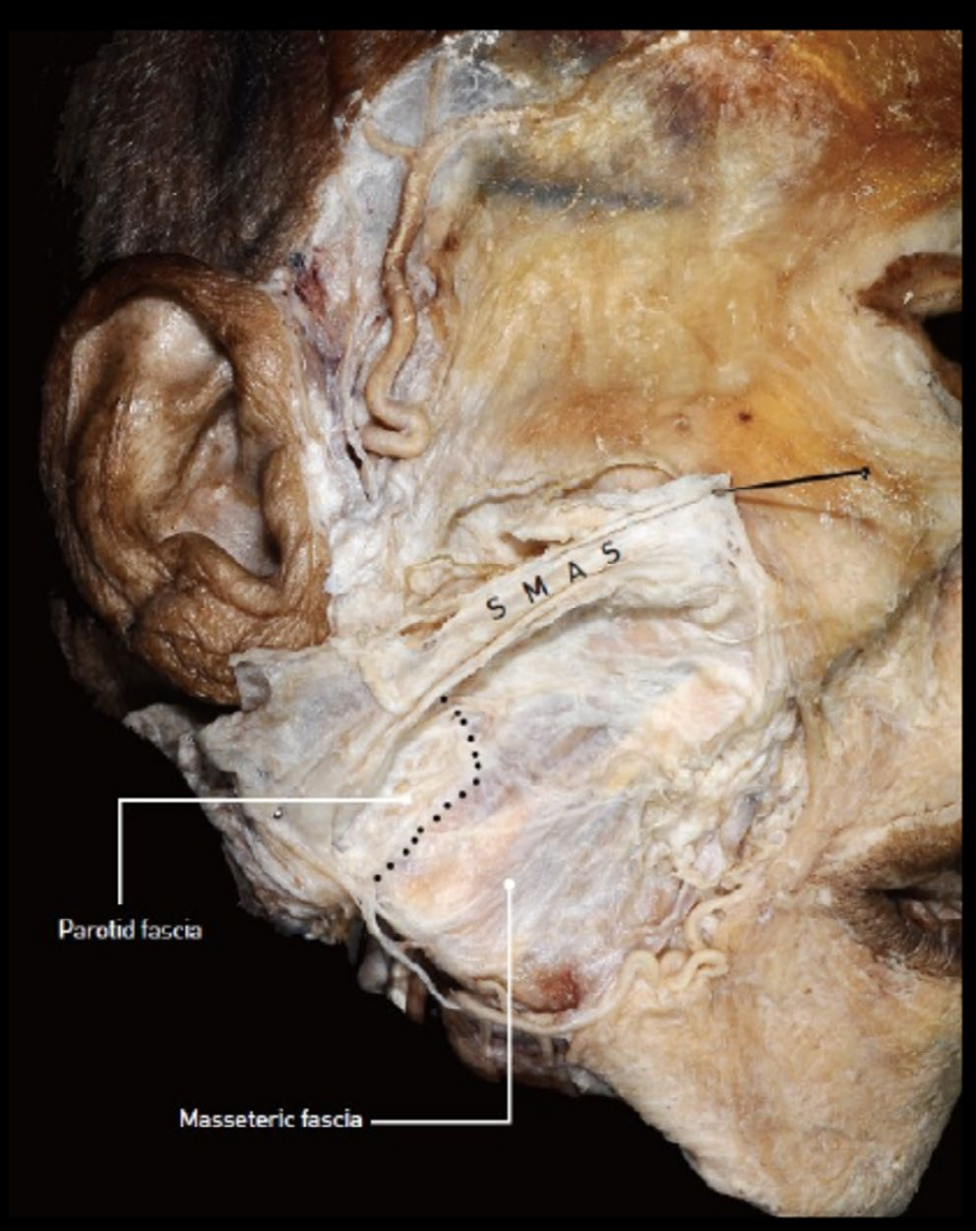
Cadaveric photograph demonstrating the relationship between the SMAS, parotid‐masseteric fascia, and parotid gland in the lateral cheek. The parotid‐masseteric fascia functions as a protective deep boundary during safe superficial or SMAS‐adjacent thread passage.

The cadaveric photograph demonstrates the relationship between the SMAS, parotid‐masseteric fascia, and parotid gland in the lateral cheek (Figure 2). This relationship supports the concept that the parotid‐masseteric fascia and parotid capsule should be regarded as “do‐not‐cross” safety boundaries during lateral cheek thread placement. In practical terms, a thread trajectory that becomes deep enough to engage or penetrate these boundaries should be considered hazardous rather than merely a deeper lifting vector.

### 3.3 Sub‐SMAS Facial Spaces: Preparotid and Premasseteric Regions

Facelift anatomy describes several sub‐SMAS potential spaces, including the preparotid and premasseteric regions relevant to the lateral cheek [12, 13]. The premasseter space is located immediately anterior to the parotid and is bounded by membranous walls and retaining ligaments. It has been described as a relatively safe dissection plane in facelift surgery because facial nerve branches lie outside its boundaries [12, 13].

**FIGURE 3:**
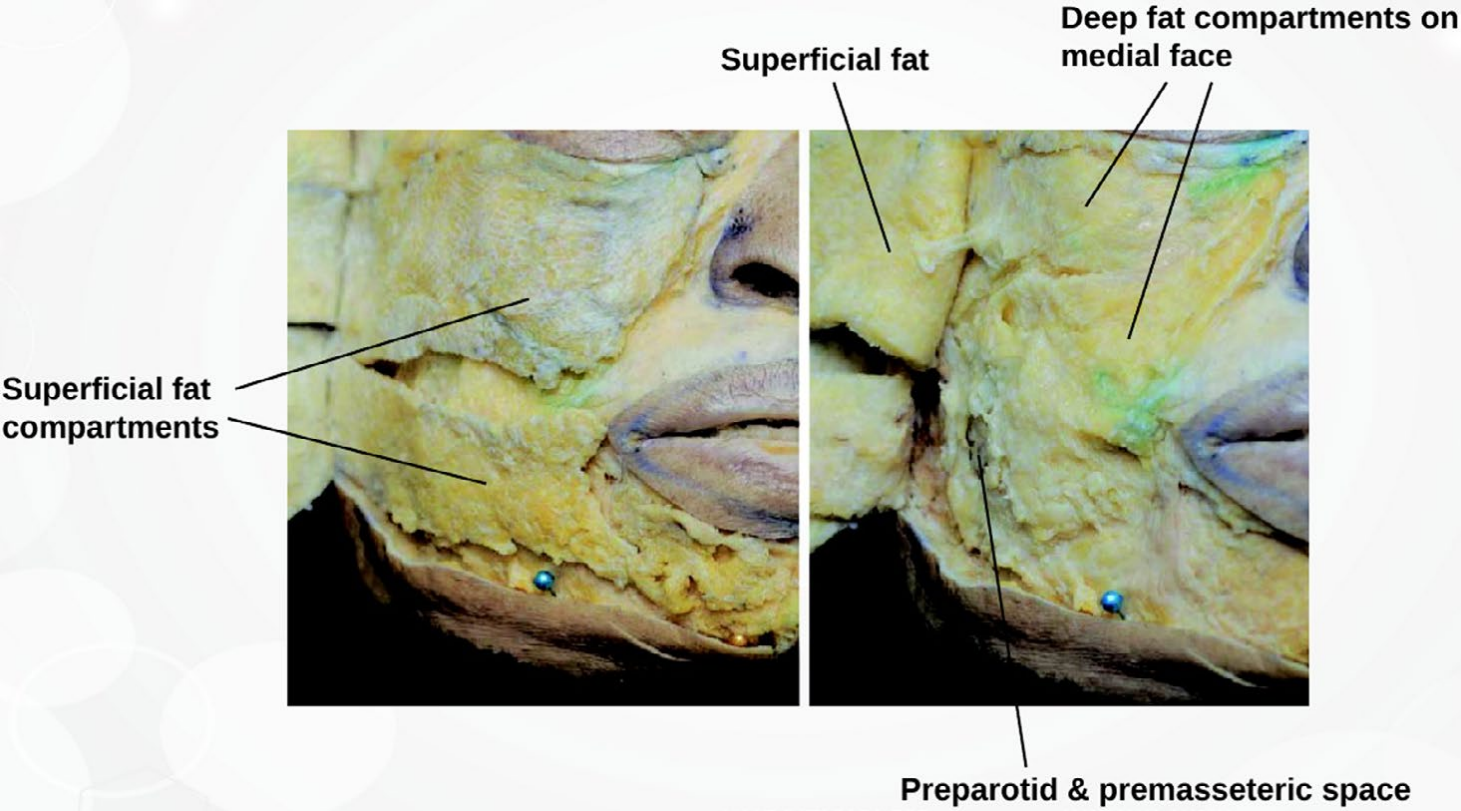
Cadaveric photographs demonstrating superficial fat compartments and the preparotid/premasseteric sub‐SMAS region of the lateral cheek. These spaces illustrate why controlled plane maintenance is required when thread trajectories pass through the preauricular and lateral cheek region.

Thread lifting does not reproduce open sub‐SMAS dissection, and a blind cannula pass should not be assumed to follow the same controlled anatomical space as surgical dissection. Nevertheless, the safety principle derived from facial space anatomy remains clinically relevant: the operator should maintain a deliberate plane and avoid deep violation of the parotid‐masseteric fascia. Cadaveric images of the preparotid and premasseteric region demonstrate how a safe corridor may be lost when the cannula is redirected forcefully after encountering resistance (Figure 3). Thus, sub‐SMAS terminology should be used cautiously in thread lifting unless the operator can reliably maintain the intended depth [15, 16, 17, 18].

### 3.4 Cadaveric Observation: Thick Lateral SMAS and Thread Position

The cadaveric demonstration shows that the lateral SMAS can form a substantial fibrous layer that may be elevated as a unit. Depending on the intended technique and depth of insertion, threads may lie within the superficial fat, adjacent to the SMAS, or in a controlled plane near the SMAS. These observations are consistent with the concept that safe thread placement depends not only on the entry point and vector but also on continuous depth control during advancement.

**FIGURE 4:**
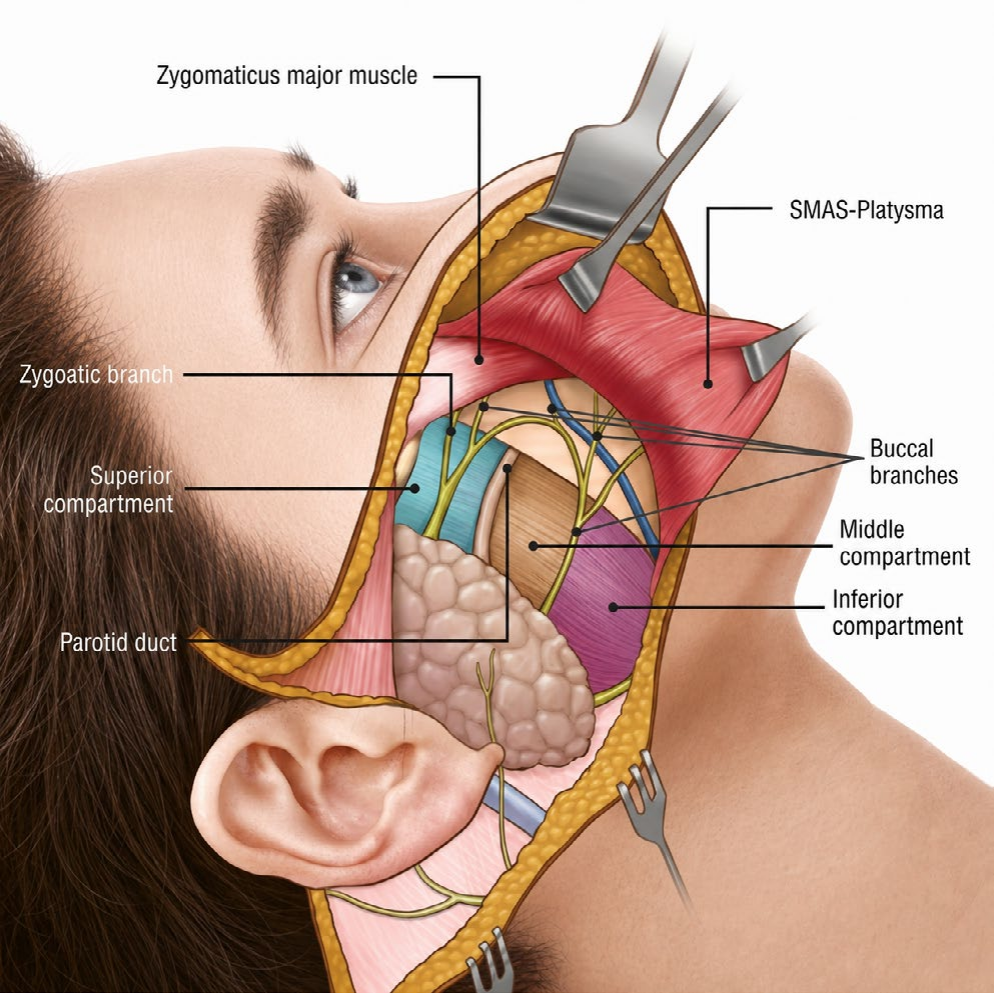
Revised cross‐sectional schematic illustration of the lateral cheek demonstrating the layered relationship among the skin, superficial subcutaneous fat, SMAS, parotid‐masseteric fascia, parotid capsule, parotid gland, parotid duct region, and facial nerve branches. The illustration distinguishes a superficial subcutaneous corridor, an SMAS‐adjacent corridor, and a hazardous deep trajectory that violates the parotid‐masseteric fascia and parotid capsule. The figure emphasizes that safe placement depends on maintaining the thread superficial to the protective fascial boundary rather than on a fixed universal depth.

Near superior transition zones such as the zygomatic arch, the planned plane may need to become more superficial because of changing fascial anatomy and zones of adherence. A fixed‐depth pass despite changing tissue resistance may produce a deeper‐than‐intended trajectory. Figure 4 illustrates the layered relationship among the skin, superficial fat, SMAS, parotid‐masseteric fascia, parotid capsule, parotid gland, facial nerve branches, and parotid duct. The figure should be interpreted as a cross‐sectional plane‐control schematic rather than as a single universal depth recommendation, because tissue thickness and parotid extension vary among patients.

### 3.5 Proposed Cadaveric Mechanism of Parotid Injury: Breach of Fascia and Capsule With Intraparenchymal Thread Passage

The cadaveric demonstration suggests a plausible mechanism for parotid gland injury during lateral cheek thread lifting. The most clinically important error occurs when the cannula passes deep to the intended superficial or SMAS‐adjacent corridor, breaches the parotid‐masseteric fascia and parotid capsule, and enters the parotid parenchyma. After skin elevation and SMAS incision in the cadaveric specimen, the thread can be seen engaging the parotid‐masseteric fascia and traversing into glandular tissue rather than remaining superficial to the protective fascial boundary.

This mechanism may be difficult to recognize clinically because parotid parenchyma can appear yellow and lobulated, resembling adjacent fat. As a result, intraglandular thread passage may not be immediately apparent during the procedure and may only become evident after postoperative swelling, sialocele, salivary fistula, meal‐related discomfort, or ductal symptoms develop. These cadaveric findings are consistent with clinical reports of salivary complications after facial esthetic procedures, but they should be interpreted as anatomical plausibility rather than definitive proof that a specific thread trajectory causes confirmed intraparotid placement in all clinical cases [8, 9].

**FIGURE 5:**
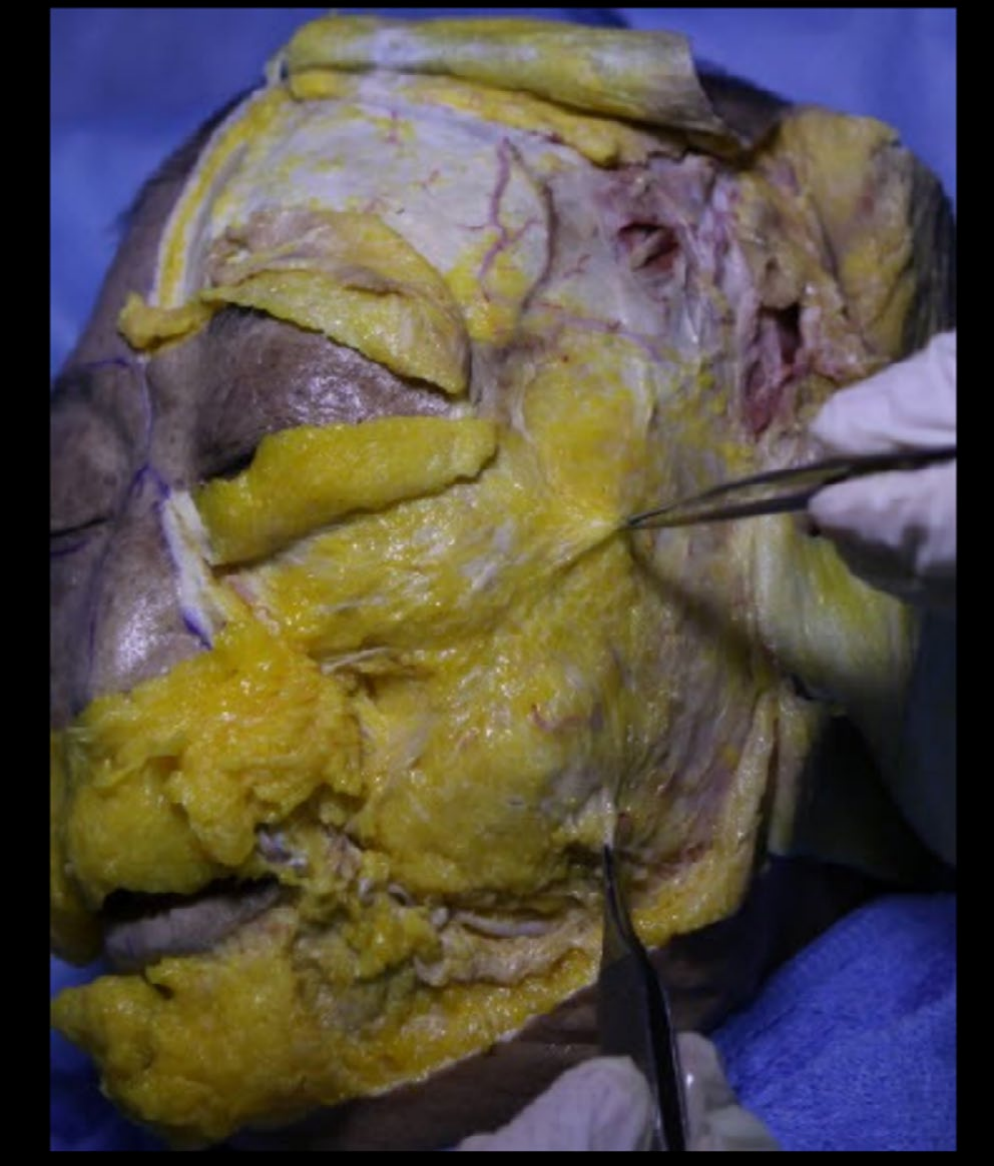
Cadaveric photograph demonstrating the relatively thick SMAS layer in the lateral facial region. The fibrous quality of the lateral SMAS may provide mechanical support for lifting but may also contribute to cannula resistance or deflection if the intended plane is not maintained.

**FIGURE 6:**
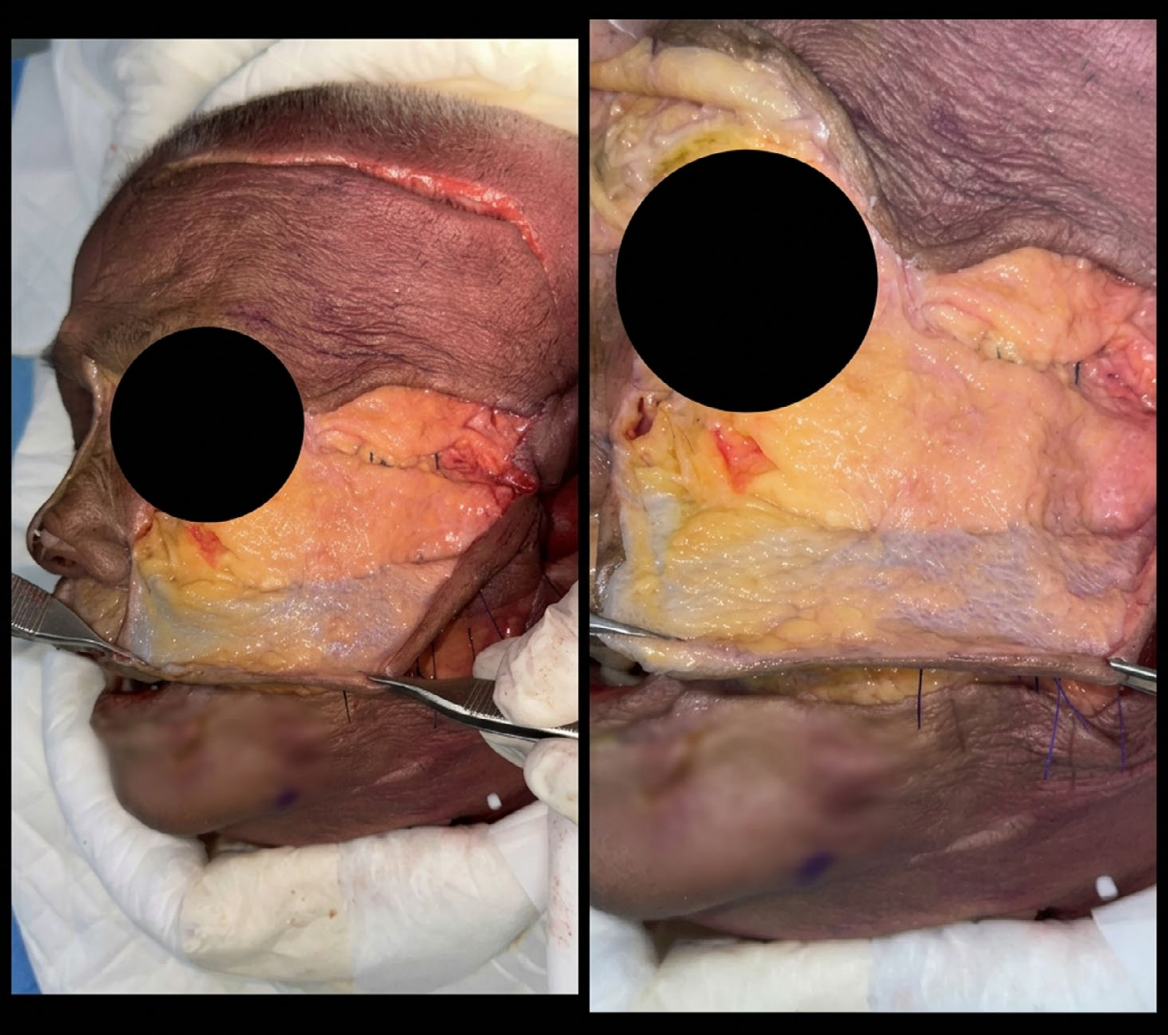
Cadaveric dissection after lateral cheek thread placement, showing threads visible beneath the elevated skin within the superficial fat layer. In superior transition zones, a deeper‐than‐intended course may occur if the operator does not adjust the plane in response to changing tissue resistance. The Sihler Thread has been used for the study.

**FIGURE 7:**
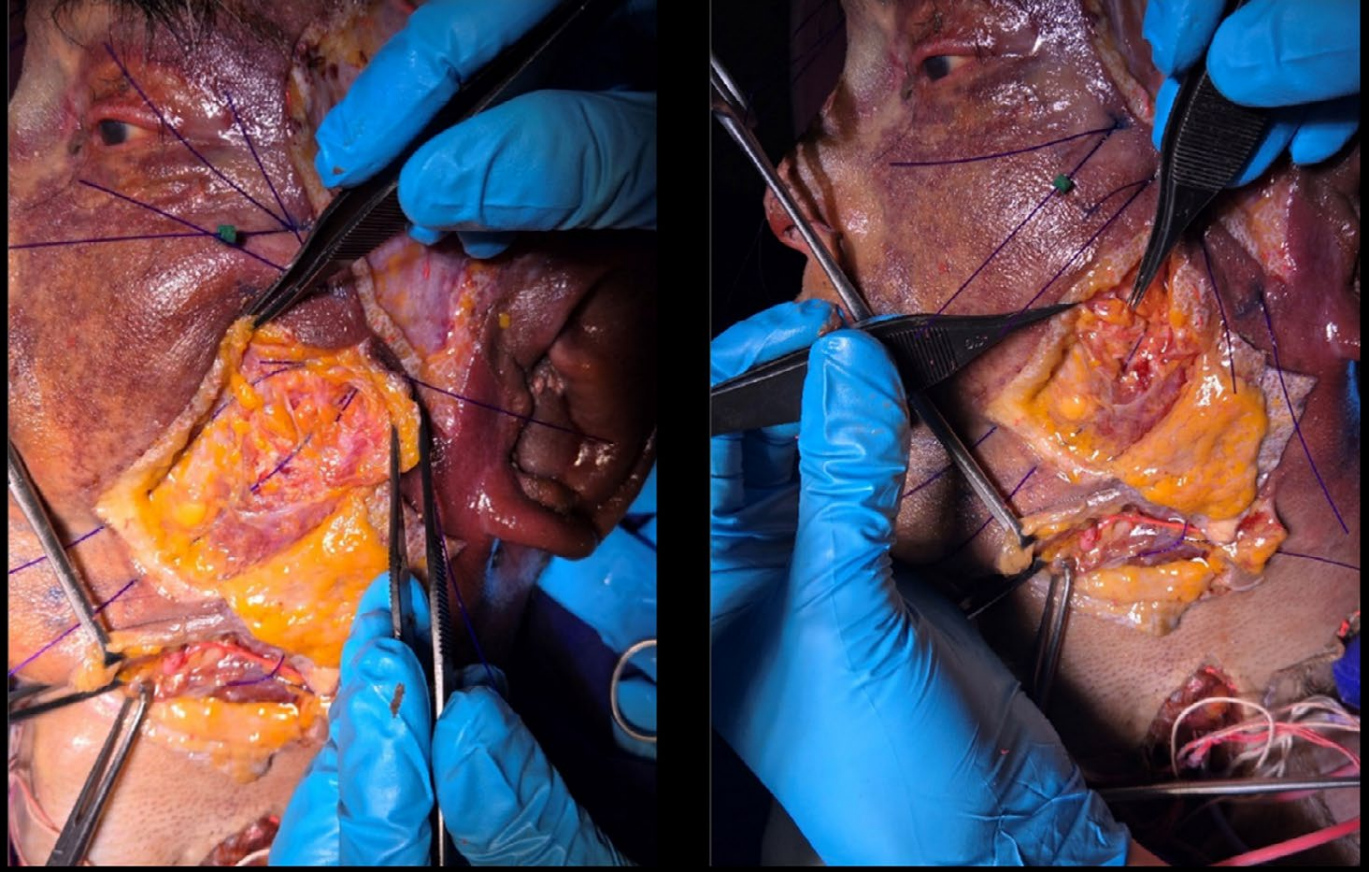
Cadaveric demonstration of the proposed parotid injury mechanism. The thread is shown traversing through the parotid‐masseteric fascia and entering parotid gland parenchyma after SMAS elevation, illustrating how deep fascial violation may result in intraglandular thread passage. This finding should be interpreted as anatomical correlation supporting a plausible mechanism rather than definitive clinical proof of causation. The Sihler Thread has been used for the study.

Figures 5, 6, 7 illustrate the progressive anatomical basis of this mechanism: the thick lateral SMAS, the relationship of the thread to superficial and deep layers, and the potential course of the thread through the parotid‐masseteric fascia into the parotid gland.

## 4 Discussion

### 4.1 Principal Findings

This anatomical technical note proposes that parotid gland injury during lateral cheek cogged thread lifting is anatomically consistent with loss of plane control and violation of protective deep fascial boundaries, particularly the parotid‐masseteric fascia and parotid capsule. The lateral cheek is characterized by a thick SMAS, strong retaining ligaments, and sub‐SMAS facial spaces that have been well described in facelift anatomy [10, 11, 12, 13, 14]. For thread lifting, these same structures may create predictable points of resistance during cannula passage. Therefore, increased resistance should prompt reassessment of the insertion plane rather than forceful advancement.

The practical implication is that safety depends on continuous plane control rather than on the entry point or vector alone. A superficial subcutaneous pass may reduce the risk of parotid violation but may carry esthetic tradeoffs, whereas a SMAS‐adjacent pass may improve tissue engagement while still respecting the parotid‐masseteric fascia. A hazardous deep pass begins when the cannula breaches the protective fascial boundary and enters the parotid capsule or parenchyma. This layered framework provides a reproducible way to translate cadaveric anatomy into procedural decision‐making.

### 4.2 Comparison With Existing Literature

The premasseter space literature is instructive because it describes a naturally occurring cleavage plane immediately anterior to the parotid, with facial nerve branches positioned outside the space [12, 13]. Although thread lifting is not equivalent to open sub‐SMAS facelift dissection, many lateral cheek thread trajectories overlap this anatomical region. The practical implication is that thread lifting can borrow the safety logic of facial space anatomy: the operator should maintain a controlled plane superficial to the parotid‐masseteric fascia, recognize when resistance suggests loss of plane, and avoid breaching the parotid capsule.

Complication studies also reinforce the importance of anatomical knowledge and technique control. In a meta‐analysis of facial thread lifting, swelling and dimpling were common, whereas severe complications were less frequent but clinically significant [4]. Large clinical series and reviews have emphasized that unfamiliarity with anatomy, inappropriate depth, and technical error contribute to thread‐lifting complications [5]. Parotid injury has been listed among early complications of PDO thread lifting [6], and comprehensive complication reviews identify parotid gland injury as a rare but challenging event. In addition, open facelift literature documents iatrogenic parotid injury resulting in fistula or sialocele, supporting the broader principle that salivary structures are vulnerable when fascial barriers are violated [8].

However, the current literature does not yet provide strong direct evidence linking a precisely defined thread trajectory to imaging‐confirmed intraparotid placement. Therefore, the mechanism proposed in this article should be understood as an anatomically plausible explanation supported by cadaveric correlation and related clinical complication reports, rather than as proven clinical causation. This distinction is important because postoperative swelling, sialocele, ductal obstruction, and salivary fistula may arise through more than one mechanism, including direct gland violation, ductal trauma, local inflammation, fibrosis, or compression.

### 4.3 Clinical Implications

The cadaveric images in this study provide an anatomical explanation for how parotid injury may occur during lateral cheek thread placement. Once the parotid‐masseteric fascia and parotid capsule are breached, the cannula or thread may pass into parotid tissue. Because parotid parenchyma can resemble adjacent fat, an intraglandular course may not be recognized immediately. This mechanism provides a plausible explanation for delayed postoperative swelling, sialocele, salivary fistula, or ductal symptoms after facial esthetic procedures [8, 9].

Intraprocedural cues suggesting loss of the intended superficial or SMAS‐adjacent corridor and recommended immediate responses are summarized in Table 2.

**TABLE 2:** Intraprocedural plane‐control cues suggesting loss of the intended superficial or SMAS‐adjacent corridor.

Cue	Likely interpretation	Suggested response
Marked resistance at a fixed point with inability to advance	Cannula tip may be engaging a retaining ligament, dense septal zone, or fibrous SMAS region	Stop forward pressure; withdraw slightly; redirect within the intended plane or select a more superficial corridor; avoid forceful thrusting
Cannula deflection or unexpected change in trajectory	The cannula may be redirected by ligamentous resistance or dense fibrous tissue	Reassess the vector and depth; do not continue blindly along a deviated path
Sudden deep “drop” after prior resistance	Possible perforation through the SMAS, parotid‐masseteric fascia, or another fascial boundary into a deeper plane	Stop immediately; reassess depth and trajectory; abandon the track if the plane is uncertain
Loss of cannula‐tip palpability in the expected plane	The cannula may have passed deeper than intended or entered a fixed deep tissue plane	Withdraw until the tip is again controlled; redirect in a clearly palpable superficial or SMAS‐adjacent corridor
Pain or atypical sharp discomfort in awake patients	Possible irritation of a nerve branch, ductal structure, parotid capsule, or deep fascial boundary	Stop; confirm position; avoid additional passes through the same track
Immediate focal swelling over the parotid or preauricular region after a pass	Possible capsular violation, salivary leakage, hematoma, or early fluid accumulation	Assess clinically; avoid further passes in the same region; consider ultrasound evaluation if swelling is focal, progressive, or persistent
Unexpected fluid expression or persistent localized fullness	Possible salivary leakage, sialocele formation, or soft‐tissue fluid collection	Stop further manipulation; document the finding; consider ultrasound assessment and specialist referral if salivary injury is suspected
Uncertainty regarding depth near the anterior parotid or masseteric region	The trajectory may be close to the parotid‐masseteric fascia, parotid duct region, or facial nerve branches	Do not proceed by force; choose a safer superficial plane or defer further passage until the anatomy is reassessed

*Note:* This table is intended to guide intraprocedural decision‐making and does not replace clinical judgment or local standards of care.

From a practical standpoint, several safety principles can be derived. First, the lateral cheek should not be approached as a uniform plane; the operator should anticipate resistance near retaining ligaments and transition zones. Second, thread passage should remain superficial, SMAS‐adjacent, or within a deliberately controlled plane rather than being advanced blindly into deeper tissue. Third, resistance during cannula passage should be treated as a warning sign, especially in the preauricular and lateral cheek region. Fourth, if the cannula depth becomes uncertain, withdrawal and redirection are safer than forceful advancement. Fifth, if deep violation is suspected, additional passes through the same trajectory should be avoided until the tissue plane is reassessed clinically or with imaging.

These intraprocedural and postprocedural responses are organized into a practical management algorithm in Table 3.

**TABLE 3:** Suggested algorithm for suspected parotid violation during or after lateral cheek thread lifting.

Step	Clinical situation	Recommended action	Rationale
1. Intraprocedural warning signs	Marked fixed resistance, cannula deflection, sudden deep drop, loss of tip palpability, unusual deep pain, or concern that the cannula has crossed a fascial boundary	Stop further advancement immediately. Withdraw the cannula to a safer level and avoid forceful passage	Continued advancement may worsen deep fascial violation or intraparotid passage
2. Immediate reassessment	The intended plane is uncertain or the cannula trajectory has changed unexpectedly	Reassess the entry point, vector, tissue mobility, and palpability of the cannula tip. Consider abandoning the track and creating a new superficial or SMAS‐adjacent corridor	Repeated passes through an uncertain track may increase the risk of parotid capsule or ductal injury
3. Early postprocedural warning signs	Focal preauricular or lateral cheek swelling, swelling that worsens during meals, persistent localized fullness, salivary leakage, pain over the parotid region, or suspected ductal obstruction	Treat the symptoms as possible salivary involvement rather than routine swelling when they are focal, progressive, recurrent, or meal‐related	Salivary complications may present after the procedure and may be mistaken for ordinary edema or bruising
4. Imaging assessment	Swelling is progressive, localized, recurrent, meal‐related, or associated with suspected fluid collection or deep thread placement	Consider high‐frequency ultrasound to assess the parotid region, fluid collection, ductal dilatation, tissue thickness, and possible deep thread position	Ultrasound may help distinguish soft‐tissue edema from sialocele, ductal involvement, or deep/intraparotid thread placement
5. Initial conservative management	Mild symptoms without progressive swelling, infection, or clear ductal injury	Observation, compression, avoidance of further local manipulation, and close follow‐up may be considered	Mild inflammatory swelling may resolve, but persistent or meal‐related swelling requires further evaluation
6. Management of suspected sialocele or salivary fistula	Persistent or recurrent fluid collection, salivary leakage, or meal‐related swelling	Consider ultrasound‐guided aspiration when appropriate, compression, antibiotics if infection is suspected, and botulinum toxin injection in selected persistent salivary collections	These measures may reduce salivary accumulation and promote resolution
7. Referral	Progressive symptoms, recurrent sialocele, suspected ductal injury, facial weakness, persistent salivary fistula, or suspected intraparotid thread placement	Refer to an experienced specialist such as an otolaryngologist, plastic surgeon, oral and maxillofacial surgeon, or clinician experienced in salivary complications	Ductal injury, facial nerve involvement, or intraparotid foreign material may require specialized evaluation or intervention

*Note:* This algorithm is intended as a practical safety framework rather than a rigid treatment protocol; management should be individualized according to symptom severity, imaging findings, and local expertise.

### 4.4 Plane‐Control Framework for Lateral Cheek Thread Insertion

For procedural purposes, plane control can be divided into three practical categories. The first is the superficial subcutaneous corridor, in which the thread remains within the fat superficial to the SMAS. This corridor generally provides the greatest distance from the parotid capsule but may increase the risk of contour irregularity, dimpling, palpability, or visibility in thin patients. The second is the SMAS‐adjacent corridor, in which the thread remains immediately superficial to or closely related to the SMAS without crossing the parotid‐masseteric fascia. This corridor may offer stronger mechanical engagement while preserving the protective deep fascial boundary. The third is the hazardous deep corridor, in which the cannula or thread violates the parotid‐masseteric fascia, parotid capsule, or glandular tissue.

This framework emphasizes that “depth” should not be interpreted as a fixed measurement applicable to every patient. Instead, depth should be understood in relation to tissue layers, resistance, patient‐specific soft‐tissue thickness, and proximity to the parotid‐masseteric fascia. The operator should reassess the plane whenever resistance increases, the cannula tip becomes difficult to palpate, the trajectory changes unexpectedly, or the tissue feels fixed rather than mobile. In such circumstances, withdrawal and redirection are safer than continued advancement.

### 4.5 Balancing Safety and Esthetic Effect

Esthetic efficacy must be balanced against anatomical risk. Superficial subcutaneous placement may reduce the likelihood of deep parotid injury because it remains farther from the parotid‐masseteric fascia and parotid capsule. However, superficial placement can increase the risk of dimpling, thread visibility, palpability, contour irregularity, or limited lifting durability, particularly in thin patients or in regions with limited soft‐tissue coverage [4, 7].

Deeper SMAS‐adjacent or selectively sub‐SMAS strategies may improve mechanical coupling, vector stability, and lifting effect by engaging more fibrous tissue. Some experienced practitioners may therefore prefer deeper trajectories for selected patients or specific thread systems. The tradeoff is that deeper placement requires more precise anatomical control and may reduce the margin of safety near the parotid gland, facial nerve branches, parotid duct, and parotid‐masseteric fascia. The risk–benefit balance may also vary among PDO threads, PLLA threads, and cone‐based systems because these devices differ in stiffness, tissue engagement, anchoring behavior, and duration of effect.

Therefore, the present article should not be interpreted as recommending a single universal depth for all thread‐lifting procedures. Rather, it emphasizes that any chosen depth must preserve the protective fascial boundary over the parotid gland. Patient‐specific factors, including prior surgery, fibrosis, facial volume, skin thickness, soft‐tissue laxity, and parotid extension, may further influence the risk of misplacement. At present, high‐quality comparative studies directly evaluating thread depth, lifting efficacy, vector durability, and salivary complications remain limited.

### 4.6 Role of Ultrasound

Ultrasound may provide an important adjunct for improving safety in high‐risk lateral cheek procedures. Preprocedural ultrasound can help identify the anterior border of the parotid gland, estimate soft‐tissue thickness, localize the parotid duct region, and recognize patient‐specific variation in gland extension or tissue fibrosis. These findings may be particularly useful in thin patients, revision cases, patients with prior facial surgery, or cases in which surface landmarks are unreliable [19, 20, 21].

Intraprocedurally, ultrasound may help clarify the tissue plane when the cannula trajectory is uncertain, although routine real‐time ultrasound guidance for every thread pass may not be practical in all clinical settings. Postprocedurally, ultrasound can assist in evaluating focal swelling, suspected sialocele, fluid collection, ductal dilatation, or a thread that appears to have been placed deeper than intended. Ultrasound may therefore bridge the gap between anatomical plausibility and clinical confirmation by documenting whether a suspected complication involves the parotid gland, parotid duct, or surrounding soft tissue.

The role of ultrasound should be considered complementary rather than a substitute for anatomical knowledge and careful technique. Its greatest value may be in risk stratification, early diagnosis of suspected salivary complications, and documentation of misplaced threads or fluid collections in anatomically high‐risk regions.

### 4.7 Suggested Algorithm for Suspected Parotid Violation

To translate these anatomical principles into clinical decision‐making, a stepwise algorithm for suspected parotid violation is summarized in Table 3. During lateral cheek thread insertion, the first step is to stop further advancement when warning signs occur, including unexpected deep resistance, sudden loss of tactile control, inability to palpate the cannula tip in the expected plane, abrupt change in trajectory, unusual deep pain, or concern that the cannula has crossed a fixed fascial boundary. The cannula should be withdrawn to a safer level rather than forced forward, and repeated passes through the same uncertain trajectory should be avoided.

If concern persists after the procedure, early assessment should focus on symptoms suggestive of salivary involvement, including focal preauricular or lateral cheek swelling, swelling that worsens during meals, persistent fluid collection, salivary leakage, pain over the parotid region, or signs of ductal obstruction. Ultrasound assessment should be considered when swelling is progressive, localized, meal‐related, recurrent, or associated with suspected fluid collection. Initial conservative measures may include observation, compression, and avoidance of further local trauma when symptoms are mild.

If a sialocele, salivary fistula, ductal injury, or intraparotid thread placement is suspected, referral to an experienced specialist should be considered. Depending on clinical findings, management may include ultrasound‐guided assessment, aspiration of fluid collection, compression, antibiotics when infection is suspected, botulinum toxin injection to reduce salivary flow, sialendoscopic evaluation, or surgical management. The algorithm in Table 3 is intended as a practical safety framework rather than a rigid treatment protocol, because management should be individualized according to symptom severity, imaging findings, and local expertise.

### 4.8 Educational and Training Implications

The mechanism proposed in this article also has implications for procedural training. Thread lifting is performed by clinicians with variable exposure to facial anatomy, cadaveric dissection, ultrasound imaging, and complication management. Because the lateral cheek contains retaining ligaments, shifting fascial planes, the parotid gland, the parotid duct, and facial nerve branches, training should emphasize layer recognition rather than vector design alone.

Cadaveric education may help clinicians understand the difference between superficial, SMAS‐adjacent, and hazardous deep trajectories. Ultrasound familiarization may further improve recognition of patient‐specific tissue thickness, parotid extension, and postoperative fluid collections. Simulation‐based teaching may also be useful for reinforcing tactile decision‐making, including when to stop, withdraw, and redirect after encountering resistance. Framing thread lifting as a procedure requiring anatomical competency may reduce the risk of preventable complications in high‐risk facial regions.

### 4.9 Limitations

This study has several limitations. First, the cadaveric component is anatomical and illustrative rather than quantitative. Therefore, the proposed mechanism should be considered hypothesis‐generating rather than definitive clinical proof. Second, cadaveric tissue does not fully reproduce the elasticity, bleeding, tissue turgor, pain feedback, and dynamic resistance encountered in living patients. Third, the literature review was targeted and narrative rather than systematic, so selection bias is possible. Fourth, the study does not include clinical outcome data, postoperative imaging confirmation, or prospective validation of parotid injury mechanisms. Fifth, because no clinical vignette with imaging‐confirmed intraparotid thread placement was included, the translational correlation remains anatomical rather than case‐based. Finally, anatomical variation among patients may influence the relationship between the thread trajectory, parotid‐masseteric fascia, parotid capsule, parotid duct, and facial nerve branches.

These limitations are important because the available evidence does not yet establish a direct causal relationship between a specific thread depth and confirmed parotid injury. The present article should therefore be interpreted as an anatomy‐based safety framework intended to guide procedural awareness, complication recognition, and future clinical validation.

### 4.10 Future Directions

Future studies should validate this proposed mechanism using clinical imaging, ultrasound‐guided anatomical assessment, prospective complication analysis, or standardized cadaveric simulation. Imaging‐confirmed case reports or small clinical series would be particularly useful for determining whether suspected thread‐related salivary complications correspond to intraparotid thread passage, ductal trauma, compression, or inflammatory obstruction. Comparative studies evaluating different insertion planes, vector designs, thread materials, and patient‐specific anatomical risk factors would help determine how best to balance lifting efficacy with avoidance of salivary and neural injury.

## 5 Conclusion

Lateral cheek cogged thread lifting traverses an anatomically complex region in which the parotid gland lies immediately deep to clinically important fascial planes. Parotid injury is anatomically consistent with loss of plane control and breach of the parotid‐masseteric fascia and parotid capsule during forceful or misdirected cannula passage. Respect for retaining ligament boundaries, deliberate maintenance of a superficial or SMAS‐adjacent corridor, avoidance of force in high‐resistance tissue, and early imaging assessment when salivary injury is suspected may reduce risk. The cadaveric correlations presented in this study should be considered anatomy‐based and hypothesis‐generating rather than definitive clinical proof. Further clinical, ultrasound‐based, and procedural validation is required.

## Author Contributions

Conceptualization: Kyu‐Ho Yi and Gi‐Woong Hong. Writing – original draft preparation: Kyu‐Ho Yi. Writing – review and editing: Kyu‐Ho Yi and Gi‐Woong Hong. Visualization: Kyu‐Ho Yi and Gi‐Woong Hong. Supervision: Kyu‐Ho Yi and Gi‐Woong Hong. All authors have reviewed and approved the article for submission.

## Funding

The authors have nothing to report. The products utilized in this study were donated by the injectors for the purposes of this study.

## Disclosure

This study was conducted in compliance with the principles set forth in the Declaration of Helsinki.

## Ethics Statement

This study used cadaveric anatomical material and did not enroll living human participants. The cadaveric material was handled in accordance with applicable anatomical donation and ethical regulations.

## Consent

Consent for use of cadaveric material was obtained through an approved body donation program. The authors have nothing to report.

## Conflicts of Interest

The authors declare no conflicts of interest.

## Data Availability

The data that support the findings of this study are available from the corresponding author upon reasonable request.
